# Advances in high-resolution imaging of centrioles and cilia

**DOI:** 10.1242/jcs.264260

**Published:** 2025-10-28

**Authors:** Olivier Mercey, Marine Brunet, Paul Guichard, Virginie Hamel

**Affiliations:** Department of Molecular and Cellular Biology, Faculty of Sciences, University of Geneva, Geneva 1211, Switzerland

**Keywords:** Cilia and flagella, Centriole, Imaging, Super-resolution microscopy, Cryo-electron microscopy

## Abstract

Centrioles, cilia and flagella are evolutionarily conserved organelles essential for a wide range of cellular functions, including division, polarity, signaling, motility and fluid flow. Despite their fundamental roles in development and disease, their small size and intricate architecture have historically limited high-resolution imaging and molecular-scale analysis. These cylindrical structures, measuring ∼200–250 nm in diameter, approach the diffraction limit of conventional fluorescence microscopy. In this Review, we highlight recent advances in imaging modalities that have significantly deepened our understanding of centriolar and ciliary structure and function. We focus on innovations in cryo-electron tomography, cryo-electron microscopy, super-resolution microscopy, atomic force microscopy and expansion microscopy, and discuss how these approaches have provided unprecedented insights into the molecular organization of these organelles, bridging the gap between cellular and molecular scales in health and disease contexts.

## Teaser

**Summary:** A review of the progressive advances in light and electron microscopy that have revealed unprecedented structural and molecular insights into centrioles and cilia, and reshaped our view of these essential organelles and their roles in disease

## Introduction

### A historical perspective on imaging centrioles and cilia

**Fig. 1.**
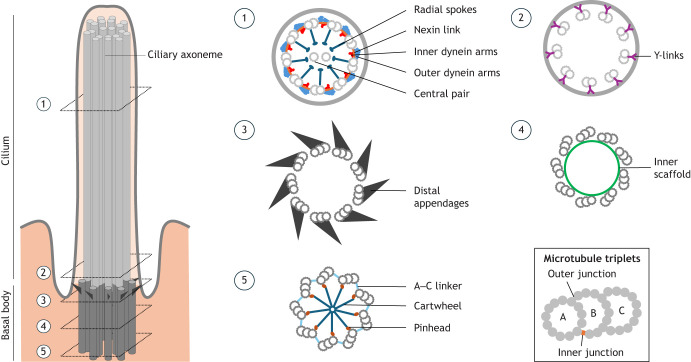
**centriolar and ciliary structures.** Schematic representations of a cilium and a basal body as a longitudinal view (left) or as diverse transverse sections (right), highlighting key structural features at different position along proximal to distal axis. On the right, an inset depicts the structure of a microtubule triplet.

Centrioles and cilia are evolutionarily conserved, microtubule-based organelles that play fundamental roles in cell biology. Because cilia are templated from centrioles, they share key structural features, most notably the characteristic 9-fold symmetry of their microtubule architecture. Along their proximal to distal axis, centriolar and ciliary microtubules are decorated with specific structural components that confer essential functional properties (Fig. 1).

Owing to this intimate structural relationship, the imaging histories of centrioles and cilia have followed parallel trajectories. However, features of cilia were often resolved earlier, a disparity largely due to the smaller length of centrioles.

**Fig. 2.**
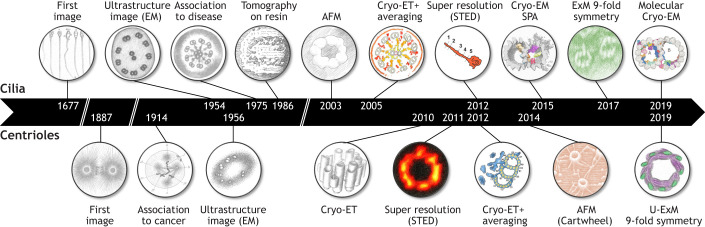
**Historical overview of cilia and centriole imaging.** Chronological timeline illustrating major advancements in imaging technologies for cilia (top) and centrioles (bottom). Spanning from the 17th century to the 2020s, progress in light and electron microscopy has profoundly expanded our structural and molecular understanding of these organelles. Images are AI-generated representations of the following papers. Cilia, left to right: Hughes and Pierson, 2013; Fawcett and Porter, 1954; Afzelius et al., 1975; McEwen et al., 1986; Seixas et al., 2003; Nicastro et al., 2005; Yang et al., 2013; Maheshwari et al., 2015; Halpern et al., 2017; Ma et al., 2019. Centriole, left to right: Scheer, 2014; Satzinger, 2008; de Harven et al., 1956; Guichard et al., 2010; Lau et al., 2011; Li et al., 2012; Pfreundschuh et al., 2014; Gambarotto et al., 2019.

The story begins in the 17th century, when Antonie van Leeuwenhoek observed on cilia and flagella on protozoa, describing them as motile structures (Fig. 2) (Leeuwenhoek, 1677). Later, in 1887, Theodor Boveri and Edouard van Beneden both determined that the polar bodies of the mitotic spindles, previously described by Walther Flemming, were permanent cellular components that appeared to function as organizing centers for cell division (Boveri, 1887; van Beneden and Neyt, 1887; Scheer, 2014). van Beneden referred to them as corpuscule central, whereas Boveri named them centrosomes, the currently used terminology. Boveri then identified a small, denser granule at the center of the centrosome and named it the centriole (Boveri, 1888). A few years later, he proposed that supernumerary centrosomes could play a role in cancer development (Fig. 2) (Boveri, 1914, 2008; Scheer, 2014).

However, imaging these structures remained a challenge, as their width size corresponds to the physical barrier of resolution for light microscopy of ∼200 nm (Abbe, 1873). Because of this, it was not until the advent of electron microscopy (EM) in cell biology in the 1940–1950s that new ultrastructural information on these organelles became available. In 1954, Fawcett and Potter described the ciliary ultrastructure from several type and species of ciliated cells (Fawcett and Porter, 1954). This work produced the first observation of the canonical 9+2 structure of motile cilia with nine doublets of microtubules (then called ‘filaments’) around one central pair (Figs 1 and 2). Less than 10 years later, Barnes realized that primary cilia lacked the central pair, thereby establishing key structural paradigms still referenced today (Barnes, 1961). In parallel, the first EM images of centriole microtubule triplets were obtained by Etienne de Harven in 1956, although they were not recognized as such at the time (de Harven et al., 1956) (Figs 1 and 2).

Throughout the second half of the 20th century, EM studies defined much of what we now know about centriole and cilium ultrastructure. Indeed, this nanoscale resolution made it possible to associate ultrastructural defects with cellular phenotypes and even disease. This was perfectly exemplified in 1975 and 1976 when Afzelius analyzed the ultrastructure of sperm axonemes (Afzelius et al., 1975) and respiratory epithelium (Afzelius, 1976) from individuals with Kartagener's syndrome, a genetic disorder classified as a cilia-related disease or ciliopathy (Fig. 2). Thanks to EM micrographs of these unusually immotile cilia, he discovered that they were lacking dynein arms, confirming his earlier hypothesis that dyneins generate axonemal microtubule gliding by attaching to one microtubule doublet and ‘walking’ along the adjacent one. This generates sliding forces between doublets that are converted into bending motions by structural constraints, producing the characteristic beating of cilia (Afzelius, 1959) (Fig. 1). A few years after, Goodenough and colleagues greatly contributed to the understanding of the motility substructures of cilia using freeze-fracture and deep-etching coupled to transmission electron microscopy (TEM) (Goodenough and Heuser, 1982, 1984, 1985). By rapidly freezing and fracturing cilia in order to visualize internal features, the authors notably revealed subtle details on dynein arm and radial spoke 3D configuration and periodicity (Fig. 1).

Although EM offered extensive ultrastructural information in cell biology, sample preparation methods, such as resin embedding and heavy atom staining, limited the retrieval of molecular information and protein identification. The emergence of immunofluorescence microscopy in the 1990s, along with the development of antibodies against specific centrosomal and ciliary proteins, as well as advances in live imaging using fluorescent reporters, led to a new era in centriole and cilia research. For the first time, researchers could localize proteins to these organelles within their cellular context. Moreover, the parallel advancement of DNA sequencing technologies marked a transformative period in biomedical research. Together, they enabled a deeper understanding of the functional consequences of disease-associated mutations by allowing researchers to directly link genetic alterations with specific protein expression patterns, cellular changes and pathological mechanisms.

In recent years, cryogenic-electron microscopy (cryo-EM) and super-resolution microscopy (SRM) have emerged as powerful high-resolution imaging techniques, revolutionizing our understanding of the molecular architecture of cilia and centrioles. A major breakthrough in the cilia field came with the application of cryo-electron tomography (cryo-ET), a technique that enables three-dimensional (3D) imaging of cellular structures in their near-native state. In 2005, Nicastro and colleagues used cryo-ET to detail the structure of axonemes from sea urchin sperm with 6 nm resolution, uncovering new insights into the molecular configuration of dynein arms (Nicastro et al., 2005) (Fig. 2). The following year, two teams extended these findings by providing 3D reconstructions of motile cilia, revealing unseen densities inside the microtubules (Nicastro et al., 2006; Sui and Downing, 2006). In 2010, the first cryo-ET study on human centrioles revealed snapshots of the microtubule triplet formation during procentriole assembly (Guichard et al., 2010) (Fig. 2). Shortly thereafter, the first 3D map of microtubule triplets at the level of centriole core was obtained in *Chlamydomonas* (Li et al., 2012) (Fig. 2). Although cryo-ET has been invaluable for visualizing macromolecular structures in their native cellular context, its resolution is limited by sample thickness and averaging constraints. In parallel, the development of single-particle analysis in cryo-electron microscopy (SPA cryo-EM) has revolutionized structural biology by enabling near-atomic resolution of isolated macromolecular complexes. Whereas cryo-ET excels at providing contextual 3D reconstructions of centrioles and cilia within intact cells, SPA cryo-EM complements it by dissecting the molecular and atomic details of individual components. The first SPA cryo-EM analysis of ciliary microtubules arose in 2015 where authors obtained a resolution of ∼19 Å (1 Å=0.1 nm), revealing how inner arm dyneins, radial spokes and microtubule inner proteins (MIPs) bind to protofilaments (Maheshwari et al., 2015) (Figs 1 and 2). Four years later, Ma and colleagues utilized SPA cryo-EM on isolated microtubule doublets to achieve a resolution <4 Å. This high resolution enabled them to model protein structures into SPA cryo-EM density maps, leading to the identification of numerous MIPs (Ma et al., 2019) (Fig. 2).

The 2010s also witnessed the rapid emergence of SRM techniques, such as stimulated emission depletion (STED) microscopy, structured illumination microscopy (SIM), and single-molecule localization microscopy (SMLM). These advancements catalyzed a paradigm shift in the molecular dissection of centrioles and cilia (see the light microscopy section under ‘Microscopy techniques applied to centriole and cilia imaging’ below). By surpassing the diffraction limit of light (∼200 nm), these methods enabled the resolution of individual protein components within centrioles and cilia at nanometer-scale precision. Centrioles were the first of these structures to be visualized with STED in 2011, revealing the nine-fold symmetry of the distal appendage centrosomal protein 164 (CEP164) (Lau et al., 2011) (Figs 1 and 2). Two years later, the first STED image of a cilium was published (Yang et al., 2013) (Fig. 2). In 2015, the invention of expansion microscopy (ExM) introduced a new dimension to super-resolution imaging by physically enlarging biological specimens to enable nanoscale visualization with conventional microscopes (Chen et al., 2015). This technique entered the cilia and centriole field more prominently by 2019, significantly enriching our molecular understanding of these organelles (Gambarotto et al., 2019; Sahabandu et al., 2019). Using ExM, the nine-fold symmetry of microtubules was resolved within cilia and later within centrioles (Halpern et al., 2017; Gambarotto et al., 2019). Since then, dozens of proteins have been mapped at the centriole level (Sahabandu et al., 2019; Kong and Loncarek, 2021; Laporte et al., 2024; Sala et al., 2024; Pudlowski et al., 2025). Atomic force microscopy (AFM), although less widely used, has also contributed. In 2003, AFM revealed the nine-fold symmetry of the ciliary axoneme (Seixas et al., 2003), and in 2014, it was used to achieve a nanoscale resolution image of the *in vitro* reconstituted centriolar cartwheel structure (Pfreundschuh et al., 2014) (Figs 1 and 2).

Despite their different principles and technical approaches, SRM and cryo-EM are remarkably complementary. Cryo-EM excels in providing native, near-atomic resolution of structural details, whereas SRM enables nanoscale localization of molecular components in cells. This Review will explore the spectrum of imaging modalities that have shaped our understanding of centrioles and cilia, highlighting examples where each technique contributed unique insights and showing how integrative approaches are poised to further illuminate these vital cellular machines. Several microscopy techniques will be detailed in this Review, emphasizing the breakthroughs each has enabled and the ways in which they complement one another. It is important to recognize that every method has its own strengths and limitations, particularly concerning the risk of artifact generation during sample preparation, image acquisition and data analysis. To ensure accurate interpretation of results, proper expertise is required to recognize these artifacts and implement strategies to minimize their formation.

## Microscopy techniques applied to centriole and cilia imaging

### EM

EM provides significantly higher resolution than light microscopy due to the shorter wavelength of electrons. Two main types of EM are commonly used – TEM and scanning electron microscopy (SEM) (Box 1).

Box 1. Microscopy techniques for structural analysis **Transmission electron microcopy (TEM):** technique that employs an electron beam to illuminate an ultra-thin section of a specimen. The image is formed from electrons that are transmitted through the sample, providing detailed information about its internal ultrastructure at the nanometer or even atomic scale.

**Scanning electron microscopy (SEM):** in contrast to TEM, this technique detects electrons scattered from the specimen's surface, enabling imaging of its topography and morphology with a resolution down to a few nanometers.

**Cryogenic electron microscopy (cryo-EM):** technique in which specimens are rapidly frozen at cryogenic temperatures (in vitreous ice) to preserve their native structure and subsequently imaged using TEM.

**Single-particle analysis cryogenic electron microscopy (SPA Cryo-EM):** cryo-EM technique that determines 3D structures of purified macromolecules. Thousands to millions of 2D projection images of individual molecules, captured in random orientations, are computationally aligned and averaged to reconstruct a detailed 3D model, often at near-atomic resolution.

**Cryogenic electron tomography (cryo-ET):** cryo-EM technique that reconstructs the 3D structure of a specimen by collecting a series of 2D images at different tilt angles. These images are computationally combined to generate a 3D tomogram with nanometer-scale resolution. The resolution can be enhanced by subtomogram averaging of repeating structures within the sample.

**Subtomogram:** within a tomogram, a subtomogram refers to a small 3D volume that has been computationally extracted to isolate a single macromolecule or repeating structural unit.

**Subtomogram averaging:** computational method in cryo-ET that enhances resolution by aligning and averaging multiple copies of identical structures within a tomogram.

**Fitting protein structure into cryo-EM maps**: protein structures are positioned within 3D density maps using computational approaches. These methods either build models directly from the protein's sequence or use predicted structures from tools like AlphaFold.

**Atomic force microscopy (AFM):** technique that scans the surface of the specimen with a tip mounted on a cantilever, detecting topographical features or mechanical forces. A laser measures the cantilever's bending and deflection, and the system create a 3D topographical map with ∼2–3 nm lateral and >0.1 nm vertical resolution.

SEM is rarely employed in cell biology as the plasma membrane hinders direct visualization of most intracellular organelles, including centrioles. However, it has been particularly valuable for studying cilia (Fig. 2) (Dalen et al., 1971; Motta and Fumagalli, 1974). For instance, Polino et al. recently used SEM with membrane extraction to produce images of submembrane ciliary structures. They clearly revealed the transition zone and ‘ciliary necklace’ as made of five to six rows of ∼12.5 nm particles spaced ∼20 nm apart around the ciliary base (Polino et al., 2023). They also reported notable morphological diversity at the distal ciliary tip, including bulbous and bulged forms. In demembranated cilia, three to seven microtubules were observed extending to the tip, where they formed a dense cap-like structure. Additionally, the authors observed a previously unrecognized submembrane feature termed the ‘ciliary ring’, which might act as a stabilizing band holding the microtubule bundle together (Polino et al., 2023).

TEM is a cornerstone of cell biology, particularly for resin-embedded samples, enabling visualization of internal cellular structures. As noted in the introduction, TEM has been pivotal in elucidating the ultrastructure of centrioles and cilia. Here, we focus on two advanced TEM-based techniques – cryo-ET and SPA cryo-EM. These methods leverage water vitrification to preserve hydrated biological samples in a frozen state, maintaining electron transparency. This approach allows imaging of biomolecules in near-native conditions. Combined with sophisticated image processing, cryo-ET and SPA cryo-EM achieve subnanometer or near-atomic resolution of molecular complexes and structures.

#### cryo-ET

**Fig. 3.**
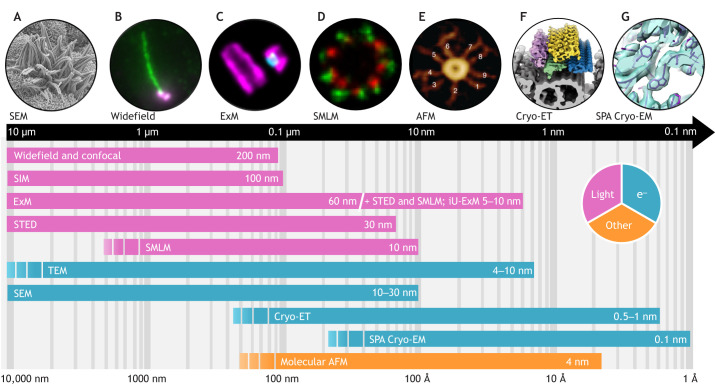
**Imaging centrioles and cilia across scales.** Overview of the spatial scales accessible to each imaging technique, including their theoretical resolution limits (logarithmic scale). (A) SEM image of mouse multiciliated trachea cells. Image was obtained by Dr Thibaut Eguether, Université and CHU Orléans, France. (B) Widefield image of a human cilium stained for acetylated α-tubulin (green) and γ-tubulin (magenta). (C) U-ExM combined with confocal imaging showing the cartwheel (Sas-6, cyan) within human procentriole; tubulin in magenta. Images in B and C were obtained in the Guichard/Hamel laboratory, University of Geneva, Switzerland. (D) STORM image of human distal appendage proteins SCLT1 (green) and CEP83 (red) (image from Yang et al., 2018 where it was published under a CC-BY 4.0 license). (E) AFM image of SAS-6 cartwheel rings (image from Banterle et al., 2021 where it was published under a CC-BY 4.0 license). (F) Cryo-ET with subtomogram averaging revealing the *Chlamydomonas reinhardtii* IFT train structure: tubulin in gray, dynein in pink, IFT-A in yellow, IFT-B1 in green and IFT-B2 in blue (image from Lacey et al., 2023 where it was published under a CC-BY 4.0 license). (G) SPA Cryo-EM structure of mouse radial spoke complex (PDB: 7DMP; Zheng et al., 2021). Note: the spatial ranges indicated for each technique are approximate and may vary depending on sample preparation and the specifications of the microscopes used.

Cryo-ET enables 3D imaging of cellular structures and macromolecular complexes at a nanometer-scale resolution. Furthermore, by applying subtomogram averaging to repeating structures, the resolution can be enhanced to reach the sub-nanometer range (Fig. 3, Box 1). Although an early attempt at 3D reconstruction of ciliary ultrastructure was made in the 1980s using thick sections of Epon-embedded newt lung (12 nm resolution; McEwen et al., 1986) (Fig. 2), the tomography era began in 2005 with the first visualization of the axoneme native ultrastructure using cryo-ET (Nicastro et al., 2005) (Fig. 2, see Introduction). Since then, further structural analyses of primary and motile cilia across multiple species unveiled details of other structural features (Kiesel et al., 2020; Leung et al., 2021; Li et al., 2022; Pinskey et al., 2022). Cryo-ET has also been a major asset in studying intraflagellar transport (IFT) complexes A and B, which mediate the transport of proteins along the axoneme for cilia assembly and function (Jordan et al., 2018; van den Hoek et al., 2022; McCafferty et al., 2022; Lacey et al., 2023; Ma et al., 2023). For example, cryo-ET combined with cryo-focused ion bean (FIB) milling has revealed the structural organization of assembling IFT trains *in situ* – one end attached to B-microtubules at the level of the transition zone, the other extending into the cytosol of *Chlamydomonas* between transition fibers (van den Hoek et al., 2022). More recently, Lacey and colleagues averaged a total of 600 tomograms of *Chlamydomonas* cilia to obtain anterograde IFT structures at a resolution reaching 9.9 Å, revealing that the IFT-B complex extends two tethers to maintain a connection with the IFT-A complex (Lacey et al., 2023). In 2024, the same group demonstrated that IFT particles undergo substantial structural reorganization during formation of the retrograde train (Lacey et al., 2024). Cryo-ET has also revealed a large number of MIPs within the microtubule doublets (Nicastro et al., 2006, 2011; Greenan et al., 2019; Legal et al., 2023; Chen et al., 2023a). However, due to the limited resolution of cryo-ET, it has generally not been possible to systematically assign specific proteins to these densities. Notable exceptions include studies combining cryo-ET with mutant and rescue experiments, which identified FAP20 at the ciliary A–B microtubule inner junction, and FAP45 and FAP52 (also known as CFAP45 and CFAP52, respectively) in *Chlamydomonas* (Dymek et al., 2019; Owa et al., 2019). Nonetheless, the molecular identity of many of these densities remains uncertain or speculative, primarily due to resolution constraints. This highlights a central limitation of cryo-ET – the challenge of unambiguously determining directly the molecular composition of complex structural elements. To overcome this challenge, cryo-ET has been combined with molecular genetics to map proteins in cilia or complemented by approaches such as protein tagging or direct antibody labeling (Pigino et al., 2011; Oda et al., 2014; Song et al., 2015; Alvarez Viar et al., 2024). More recently studies have integrated AlphaFold2-predicted structural models with fitting into cryo-ET density maps, enabling *de novo* identification of proteins (Chen et al., 2023b; Tai et al., 2023; Zhu et al., 2025). However, this hybrid approach generates only atomic models constrained by prediction and might miss fine structural details that can be uniquely resolved by SPA cryo-EM.

In 2010, cryo-ET of isolated centrioles from KE37 cells revealed that microtubule triplets in human cells initiate from A-microtubules, which are capped by the γ-tubulin ring complex (γ-TuRC), elongate distally around the cartwheel and template bidirectional growth of B- and C-microtubules, which mature independently before cap removal (Guichard et al., 2010) (Figs 1 and 2). In 2011, cryo-ET coupled to sub-tomogram averaging of *Chlamydomonas* basal bodies resolved the microtubule triplet structure within the central core region at 33 Å resolution. This analysis revealed non-tubulin densities and a Y-shaped linker at the A–B microtubule inner junction, which was later refined as part of the inner scaffold connecting the nine triplets (Li et al., 2012; Le Guennec et al., 2020) (Figs 1 and 2). Using similar approaches, structures from various species were resolved at comparable resolutions (Leung et al., 2021; Khanal et al., 2021), uncovering the native architecture of conserved elements such as the A–C linker (Li et al., 2019; Klena et al., 2020), the pinhead (Nazarov et al., 2020; Klena et al., 2020) and the cartwheel, which is composed of a 20 nm central hub, stacked rings and nine radial spokes with minor species-specific variations (Guichard et al., 2012, 2013; Klena et al., 2020; Nazarov et al., 2020) (Fig. 1). By 2019, the *Chlamydomonas* A–C linker was resolved with a 23 Å resolution, revealing WD40 β-propeller like densities (Li et al., 2019). More recently, sub-nanometer resolutions, reaching up to 6 Å, have been achieved and coupled with AlphaFold2-predicted protein structure models. This advancement enables the direct identification of proteins within the proximal and central core regions of the inner junction (Fig. 1). Notably, protein of centriole 1 (POC1) has been localized at the inner junctions of the A–B and B–C microtubules in *Tetrahymena*, where it acts to promote triplet microtubule integrity and connectivity (Ruehle et al., 2024; Li et al., 2025).

#### SPA cryo-EM

SPA cryo-EM determines 3D structures of purified macromolecules at near-atomic resolution (Fig. 2, Box 1).

Cryo-ET of cilia, flagella and centriole or basal body structures has revealed many MIPs and microtubule-associated proteins (MAPs) that periodically decorate doublet or triplet microtubules. Although a few proteins have been identified through integrative approaches combining cryo-ET with mutant and rescue experiments, most remained uncharacterized until SPA was used. In 2019, Ma et al. used biochemical methods and SPA on isolated microtubule doublets from *Chlamydomonas* axonemes, achieving a 3.4 Å resolution. They identified 33 MIPs forming a 48-nm repeat structure within the microtubule doublets and provided a detailed model of their positions, structures and interactions (Ma et al., 2019). This study marked a turning point in the study of cilia molecular architecture. Since then, SPA has been used to map the position of specific proteins within subciliary structures across several species, including *Tetrahymena*, mammals and trypanosomatids (Kubo et al., 2023; Gui et al., 2021a,b; Walton et al., 2023; Leung et al., 2025; Xia et al., 2025; Doran et al., 2025). These structures include the radial spokes and their associated complexes (Gui et al., 2021b; Grossman-Haham et al., 2021) and the ciliary central apparatus (Gui et al., 2022; Han et al., 2022). They also encompass the IFT complexes (Meleppattu et al., 2022; Hesketh et al., 2022) and the structure of the sperm flagellum across diverse species, including sea urchins, bovine, mouse and humans (Leung et al., 2023, 2025; Zhou et al., 2023; Zhao et al., 2025). Altogether, these studies have revealed extensive molecular and structural diversity of the microtubule doublets, but also unique structural features, such as a mammal-specific luminal tektin protein complex in bovine trachea cilia, likely providing additional mechanical stability during beating (Gui et al., 2021a).

Although these recent advances in SPA have significantly contributed to revealing the molecular architecture of cilia and flagella, its application to centriolar proteins remains technically challenging and has not yet been achieved. In contrast, advances in light microscopy now allow precise localization of proteins, offering a level of resolution sufficient to link them to structural features of centrioles.

### Light microscopy

In light microscopy, the resolution refers to the minimum distance at which two points can be distinguished. It is limited by light diffraction, which causes the image of a point in a microscope to appear as a blurry spot, described by the point spread function (PSF, Box 2). The resolution limit is given by Abbe's formulas: d=λ/2NA (lateral) and d=2λ/NA^2^ (axial), where λ is the wavelength and NA is the numerical aperture. Conventional light microscopes achieve ∼250 nm lateral and ∼500–800 nm axial resolution, which is insufficient for detailed imaging of centrioles and cilia. SRM, developed in the early 2000s, has bridged this gap, offering resolution complementary to EM and enabling detailed studies of centriole and cilia protein composition and biogenesis. This section introduces key SRM techniques and the contributions these have made to understanding these organelles.

Box 2. Super-resolution optical microscopy techniques **Point spread function (PSF):** describes the response of an imaging system to a point source of light (object). Instead of producing a perfect point, the system forms a blurred spot due to diffraction. The diffraction pattern, known as the Airy disk, features a central bright region surrounded by concentric rings. The size of this pattern determines how closely two objects can be positioned before their images begin to overlap, which corresponds to the resolving power of the microscope.

**Structured illumination microscopy (SIM):** widefield-based technique that consists of illuminating the sample with striped patterns, generating a moiré effect that encodes high-resolution information. Multiple images are captured with varying pattern angles and positions, then computationally reconstructed to achieve ∼100 nm lateral and 300 nm axial resolution.

**Stimulated emission depletion microscopy (STED):** confocal-based technique that consists of depleting fluorescence at the periphery of the focal spot, thereby sharpening the PSF and producing images with ∼20–50 nm lateral resolution. It uses two overlapping lasers: the first excites the fluorophores, causing them to emit photons, whereas the second donut-shaped laser stimulates the excited fluorophores in the surrounding area to return to their ground state without emitting light.

**Single-molecule localization microscopy (SMLM):** widefield-based techniques that localize individual fluorescent molecules by stochastically activating sparse subsets, ensuring that their emissions do not overlap, and fitting their PSFs before the molecules switch off. By repeating this process thousands of times, these techniques reconstruct images with ∼10–20 nm lateral and 50 nm axial resolution.

**Photo-activated localization microscopy (PALM):** SMLM technique that uses photoactivatable fluorescent proteins.

**Stochastic optical reconstruction microscopy (STORM):** SMLM technique that uses organic dyes.

**Expansion microscopy (ExM):** technique that physically enlarges biological samples by embedding them in a swellable polymer, allowing nanoscale structures to be imaged with conventional microscopes. Several protocols are available. Briefly, it consists of cross-linking the specimen to a swellable polymer and, after releasing mechanical constraints, expanding the specimen–polymer isotropically in water by a factor of 4 to 16, depending on the protocol. It can also be combined with SRM (such as SIM, STED, STORM etc.) to further increase the resolution.

**Minimal photon flux (MINFLUX):** technique combines concepts from both SMLM and STED, achieving extremely high spatial resolution with minimal photon use.

#### SIM

SIM uses patterned illumination to exceed the diffraction limit (Fig. 2, Box 2).

In 2012, SIM was applied to investigate centrosome architecture, focusing on the pericentriolar material (PCM) network that surrounds centrioles and supports the ability of the centrosome to nucleate microtubules (Mennella et al., 2012; Lawo et al., 2012; Fu and Glover, 2012; Sonnen et al., 2012). Previously described as amorphous, PCM was revealed by SIM to be organized into concentric rings of proteins surrounding the centriole wall in a progressively larger toroidal pattern, with two main layers – one adjacent to the centriole and a more extended layer, resembling a matrix. SIM further showed that *Drosophila* and human pericentrin (PCNT) localized near the centriole wall, with a radial distribution – the C-terminus positioned internally and the N-terminus externally, with peak localization ∼60 nm apart (Mennella et al., 2012; Lawo et al., 2012). Beyond centrosomes, SIM has been employed to visualize 15 evolutionarily conserved basal bodies and transition zone proteins in *Drosophila* male germ cells and sensory neurons, revealing differences across and within cells during development (Jana et al., 2018). It has also been used to study the molecular architecture of the basal foot, an appendage thought to be derived from subdistal appendages that anchor the cilia to the microtubule cytoskeleton. Using SIM, Nguyen and colleagues demonstrated that basal feet are organized into three main regions, exhibiting a similar protein composition in primary and motile cilia, but with differences in their spatial distribution (Nguyen et al., 2020). In mouse tracheal multiciliated cells, SIM resolved the ring-like arrangement of NPHP1 at the base of the axoneme, although it lacked the resolution to detect the punctate organization later captured by STORM (described below) (Shi et al., 2017). However, SIM proved particularly valuable for quantifying fluorescence intensity of multiple transition zone proteins in fibroblasts from individuals with Joubert syndrome, as its high-throughput capability makes it well-suited for large-scale data acquisition and comparative analysis (Shi et al., 2017).

#### STED

STED overcomes the diffraction limit by depleting fluorescence in the periphery of the focal spot (Fig. 2, Box 2).

This technique has played a pivotal role in elucidating how proteins are organized at the appendages and within the transition zone. STED was first applied in 2011 to uncover the nine-fold symmetry of the distal appendage protein CEP164 in murine cells (Lau et al., 2011), showing that CEP164 formed a ∼350 nm diameter ring at the distal end of centrioles, composed of nine symmetrically arranged ∼60 nm clusters (Lau et al., 2012). Later studies mapped transition zone proteins [transmembrane protein 237 (TMEM237), Abelson helper integration site 1 (AHI1), Chibby 1 (CBY1), oral-facial-digital syndrome type I (OFD1) and serologically defined colon cancer antigen 8 (SDCCAG8)], revealing restricted distal rings with diameters ranging from ∼210–270 nm, (listed in ascending order) and ninefold symmetry for OFD1 and SDCCAG8 (Lee et al., 2014). Yang and colleagues used STED to construct a spatial map of the transition zone in human primary cilia by measuring the lateral distribution and distance of several key proteins from the distal edge of the basal body marker centrin. From the innermost to the outermost layers, the proteins identified were RPGRIP1L, MKS transition zone complex subunit 1 (MKS1), TMEM67 and tectonic family member 2 (TCTN2). By aligning the positions of these proteins with EM images, the study revealed that the Y-links likely span the region where RPGRIP1L and MKS1 are localized, whereas TMEM67 and TCTN2 are positioned near the ciliary necklace (Yang et al., 2015). More recently, STED microscopy has been applied to map the distribution of phosphoinositides, membrane-associated signaling lipids, within cilia in mouse and human cells. This study revealed that phosphatidylinositol 4,5-bisphosphate [PI(4,5)P_2_] and phosphatidylinositol (3,4,5)-trisphosphate [PI(3,4,5)P_3_] form ring-like structures at the inner membrane of the transition zone and are specifically concentrated in its most proximal region, near the distal appendages (Conduit et al., 2021). STED has also been employed to study the organization of the linker connecting the two centrosomes in interphase, revealing staggered rootletin proteins spaced ∼35–40 nm apart, with CEP68 periodically associating every ∼75 nm along the filamentous structure (Vlijm et al., 2018).

#### SMLM

SLML localizes individual fluorescent molecules to reconstruct images beyond the diffraction limit. SMLM encompasses both PALM and STORM (Fig. 2, Box 2).

In 2011, SMLM was first applied to map centriolar proteins as a proof of concept (Sillibourne et al., 2011). In this study, the authors notably demonstrate that centrosomal protein 89 (CEP89, occasionally referred to as centrosomal protein 123) localizes to the mother centriole distal appendages. CEP89 forms two angled bars extending from the outer microtubule wall of the centriole (∼134 nm long, 133.5° angle) in longitudinal view and a ring structure in top view (internal diameter ∼226 nm, external ∼495.5 nm), consistent with EM previous observations (Sillibourne et al., 2011). Several studies using STORM microscopy have since refined our understanding of the molecular architecture of distal and subdistal appendages (Yang et al., 2018; Bowler et al., 2019; Chong et al., 2020) and showed that the IFT protein 88 localizes within the gaps between the blades of the distal appendages, providing insights into the spatial organization of IFT trains (Yang et al., 2018). Similarly, Hazime et al. used STORM to study the ciliary base in *Tetrahymena thermophila*, mapping nine IFT proteins and transition zone components. All IFT proteins showed nine-fold symmetry over a 30 nm axial span. IFT-B localized 139–162 nm from the center, whereas IFT-A occupied a slightly more peripheral, narrower range of 160–166 nm, with minimal overlap (Hazime et al., 2021). Additionally, this technique has been used to determine the localization of Bardet–Biedl syndrome protein 5 (BBS5) within the sensory rod cilium of mouse retinas. The results showed that BBS5 forms clusters around the connecting cilium axoneme, extending over 200 nm from its center, and that this localization remains unaffected in mice lacking other BBS proteins (Robichaux et al., 2019). STORM has also been employed to study the mechanisms of centriolar spindle assembly abnormal protein 6 (SAS-6, or SASS6 in verterbrate) cartwheel assembly, revealing that it initially forms a transient toroidal structure (∼216 nm radius) at the base of the mother centriole before S phase. During S phase, this torus condenses into a ∼60 nm focus, the nascent cartwheel, which later expands to ∼150 nm by late G2. STORM also visualized the C-terminus of SAS-6 within the cartwheel, showing a ninefold symmetric ring (Keller et al., 2014).

#### ExM

ExM was first developed by Boyden and colleagues in 2015 as a novel approach to bypass the diffraction limit of light microscopy (Chen et al., 2015). By physically enlarging biological specimens by ∼4 to 5-fold, ExM enables nanoscale imaging using standard microscopy platforms, without high-end instrumentation (Fig. 2, Box 2).

Since its introduction, several ExM variants have been developed. Label-retention expansion microscopy (LR-ExM) (Shi et al., 2021), ultrastructure expansion microscopy (U-ExM) (Gambarotto et al., 2019) and centriole-MAP (cMAP) (Sahabandu et al., 2019) all derive from the magnified analysis of the proteome approach (Ku et al., 2016). U-ExM and cMAP preserve the molecular ultrastructure of organelles like centrioles and cilia by replacing enzymatic digestion with heat-induced denaturation, followed by post-expansion immunolabeling. This modification enhances antibody compatibility while maintaining fine structural detail. Validation studies have shown that both methods closely recapitulate centriole morphology and dimensions comparable to those obtained by EM. Unlike STORM, which can struggle to resolve substructures like the nine-fold microtubule symmetry of centrioles, U-ExM and cMAP enable clearer visualization using conventional or super-resolution systems (e.g. confocal microscopy, deconvolution or STED) (Fig. 2). For example, U-ExM has been used to resolve the central pair of microtubules in the *Chlamydomonas* flagellum, revealing distinct polyglutamylation patterns between the central and outer doublets (Gambarotto et al., 2019). This technique has also facilitated the identification and spatial organization of centriolar components, including the inner scaffold [e.g. POC5, POC1B, FAM161A, WDR90, centrin-2, coiled-coil domain containing 15 (CCDC15); Le Guennec et al., 2020; Arslanhan et al., 2023; Sala et al., 2024], the A–C linker [e.g. WDR67, CCDC77, migration and invasion inhibitory protein (MIIP) (Laporte et al., 2024; Bournonville et al., 2025)] and microtubule wall-associated proteins such as Mdm1 nuclear protein (MDM1) (Sala et al., 2024). Moreover, U-ExM has been employed in large-scale analyses involving thousands of centrioles in order to reconstruct their molecular architecture using markers for 24 different substructures (Laporte et al., 2024).

Beyond cultured cell layers, U-ExM has been used to study centrioles and cilia in tissues and across multiple species, such as mouse, canine and human retina (Mercey et al., 2024; Arsenijevic et al., 2024; Takahashi et al., 2025), human kidney (Langner et al., 2024), human pancreas (Müller et al., 2024), mouse olfactory sensory neurons (Ching et al., 2022), zebrafish (Steib et al., 2022), *Drosophila melanogaster* (Brunet et al., 2025), *Caenorhabditis elegans* (Woglar et al., 2022; Pierron et al., 2023; Pfister et al., 2025) and *Naegleria gruberi* (Woglar et al., 2025)*.* Resolution can be pushed even further by combining ExM with other SRM. For example, coupling ExM with STED achieves lateral resolution down to ∼15 nm and ∼6 nm with STORM (Fig. 2) (Gambarotto et al., 2019; Bezler et al., 2022; Woglar et al., 2022; Zwettler et al., 2020; Chang et al., 2023). More recently, iterative ultrastructure expansion microscopy (iU-ExM) has been developed, enabling up to 16-fold physical expansion. This approach allows molecular mapping with ∼10 nm resolution when combined with confocal microscopy and has revealed the periodic organization of CEP290 along the connecting cilium of the mouse retina (Louvel et al., 2023).

#### AFM

AFM is a technique that physically scans the surface of specimens to produce detailed images with nanometer-scale resolution (Fig. 2).

In 2003, AFM was used to study cilia regeneration in *Tetrahymena*, revealing ∼300 nm crater-like structures with nine globular features matching the microtubule arrangement of the axoneme (Seixas et al., 2003). Later, several studies employed AFM to examine *in vitro* assembly of *Chlamydomonas reinhardtii* SAS-6, revealing that it self-assembles into cartwheel-like oligomers in ∼106 s, forming 7-, 8-, 9- and 10-fold symmetries (Pfreundschuh et al., 2014; Hilbert et al., 2016; Nievergelt et al., 2018; Banterle et al., 2021). These studies confirmed sequential SAS-6 homodimer addition and revealed alternative assembly pathways, like multimeric subunit fusion and the conformational transformation of interlinked half-rings into complete rings (Nievergelt et al., 2018). They also revealed that cartwheel formation is strongly favored on surfaces rather than in solution, offering insight into why assembly occurs on the torus structure *in vivo* but not freely within the cytosol (Banterle et al., 2021). AFM has also been instrumental in studying the motion of sea urchin sperm flagella by measuring the lateral twisting of cantilevers, the stiffness and forces exerted by cilia in amphibians and mammals, as well as the mechanical properties of polycystin-1, a protein associated with polycystic kidney disease (PKD) (Sakakibara et al., 2004; Teff et al., 2007; Droguett et al., 2017; Do et al., 2021; Forman et al., 2005; Ma et al., 2009). Despite its capabilities, AFM is not widely used in cell biology because intracellular structures are largely inaccessible due to the presence of plasma membrane. However, Usukura and colleagues have developed a protocol for AFM imaging of tissue sections that is compatible with immunofluorescence. Using this method, they visualized detailed features such as ciliary necklaces on the connecting cilium of frog photoreceptor cells and introduced a correlative imaging technique called correlative atomic force and light microscopy (CALM), which enables precise molecular localization on structures identified by AFM (Usukura et al., 2017).

## Imaging modalities to unravel cilia-associated disease mechanisms

From the early days of EM in the 1950s to the advent of cryo-EM and super-resolution techniques this past decade, each technological advancement has offered precious insights into structural and molecular mechanisms underlying cilia- and centriole-associated diseases. Because these organelles are widespread in all tissues, associated disorders are often multisyndromic, like Bardet–Biedl syndrome (BBS), Joubert syndrome (JS), Meckel–Gruber syndrome (MKS), Alström syndrome and cranioectodermal dysplasia (CED), as well as primary ciliary dyskinesia (PCD). However, a few pathologies can also be organ specific, like nephronophthisis (NPHP) and polycystic kidney disease (PKD), touching uniquely the kidney, subtypes of retinitis pigmentosa (RP) and Leber congenital amaurosis (LCA), which affects only the eye, or microcephaly (MCPH) in the brain. In this section, we highlight key advances in elucidating the structural and molecular mechanisms underlying two types of cilia-associated diseases (referred as ciliopathies), made possible through the application of advanced imaging technologies. We also discuss emerging challenges in the field and how continued innovation in imaging modalities is poised to play a pivotal role in addressing these unresolved questions.

### Deciphering ciliopathy mechanisms with advanced imaging

#### Primary ciliary dyskinesia

This group of disorders encompasses diseases resulting from defects in ciliary motility, leading to clinical manifestations in tissues that rely on motile cilia, such as chronic respiratory infections and infertility. At the ultrastructural level, these pathologies are attributed to direct or indirect impairments in components essential for ciliary motion, including the outer and inner dynein arms, the nexin–dynein regulatory complex, radial spokes and the central pair apparatus (Leigh et al., 2019).

Early ultrastructural studies using TEM laid the anatomical foundation for subsequent physiological and pathological investigations. As outlined in the Introduction, clinical manifestations such as sperm immobility had already been linked to dynein arm defects observable by TEM as early as the 1970s, decades before the causative genes were identified (Afzelius et al., 1975; Afzelius, 1976). Although the role of dynein in the sliding mechanism of microtubules in motile cilia had been already postulated at that time (Warner and Mitchell, 1980), it was first with the use of freeze-fracture and deep-etch methods, and more recently the advent of cryo-ET that classical EM data could be reinterpreted. These techniques revealed the periodic arrangement of radial spokes and dynein arms, reshaping our understanding of how defects in these structures impair ciliary motility and contribute to the pathogenesis of PCD (Nicastro et al., 2005, 2006; Goodenough and Heuser, 1982, 1985). In 2014, Lin and colleagues applied cryo-ET to compare airway cilia from healthy individuals and individuals with PCD harboring pathogenic variants in radial spoke head component 1 (*RSPH1*) (Lin et al., 2014). This analysis revealed the absence of the distal head-containing regions of radial spokes 1 and 2 (RS1 and RS2) in the cells from the individuals with PCD. More recently, Gadadhar et al. have demonstrated the crucial role of tubulin post-translational modifications (particularly glycylation) in maintaining dynein arm conformation in mouse spermatozoa using cryo-ET. This study provided mechanistic insight into how mutations affecting glycylation enzymes result in structural defects of sperm flagella, leading to loss of motility (Sudarshan et al., 2021).

Another striking advance in PCD imaging is due to the rise of cryo-EM imaging with SPA, which has enabled near-atomic resolution of dynein arm complexes. This approach has allowed detailed speculation on how specific genetic mutations might impair dynein function, thereby deepening our understanding of the mechanistic underpinnings of PCD. In 2019, Lacey et al. resolved the molecular structure of the inner dynein arm component DNAH7, demonstrating how its conformation might influence microtubule curvature during ciliary motion (Lacey et al., 2019). Two years later, three independent research groups elucidated the structures of outer dynein arm (ODA) subunits (variants in which are the major cause of PCD) marking a significant advancement in our structural understanding of this disease (Kubo et al., 2021; Walton et al., 2021; Rao et al., 2021). Importantly, in 2023, Walton and colleagues analyzed respiratory cilia from four individuals with PCD using cryo-EM. They demonstrated that the absence of specific docking factors (which mediate ODA complex attachment to microtubules) disrupts the periodicity of microtubule doublet-associated structures, thereby compromising ciliary motion (Walton et al., 2023). In a complementary approach, Brody and colleagues employed proteomics and SPA cryo-EM to investigate the impact of *CCDC39* and *CCDC40* mutations, which are associated with particularly severe PCD phenotypes. Their findings revealed not only mislocalization of ∼90 axonemal proteins but also significant ultrastructural alterations in the microtubules of cilia from affected individuals (Brody et al., 2025).

### Cilia-related retinopathies

The photoreceptor outer segment is a highly specialized derivative of the primary cilium, characterized by hundreds of stacked membranous discs that harbor light-sensitive pigments that are essential for phototransduction. This elaborate structure, which can reach lengths of up to 50 µm in humans, is crucial for visual function. Mutations in a wide range of cilia-associated genes have been implicated in retinal ciliopathies, often leading to progressive retinal degeneration and vision loss. A key region commonly affected is the connecting cilium, a specialized compartment analogous to the transition zone, that serves as a structural and functional bridge between the inner segment and the disc-bearing outer segment.

Unlike accessible tissues, such as the airway epithelium, the human retina poses significant challenges for studying ciliopathies due to its inaccessibility in living individuals. Consequently, much of our current knowledge is based on post-mortem human samples and animal models. Although the ultrastructure of the connecting cilium was described by EM as early as the late 1950s, the first report of structural abnormalities under pathological conditions came in 1986, when Hunter and colleagues compared transverse sections of the connecting cilium in healthy and Usher syndrome individuals (Usher syndrome causes retinitis pigmentosa, an eye condition that leads to progressive vision loss due to the gradual degeneration of retinal photoreceptors), revealing microtubule defects (Hunter et al., 1986). Two decades later, Nickell et al. applied cryo-ET to retinal tissue, generating the first 3D reconstructions of outer segment disc architecture (Nickell et al., 2007), and in 2012, Wensel's group used cryo-ET in mouse models of inherited retinal degeneration to investigate nanoscale organization of the connecting cilium (Gilliam et al., 2012). The same team subsequently combined EM with STORM to delineate the molecular organization of key ciliary components, which proved particularly informative for studying mutations in *CEP290*, one of the genes most frequently found to have pathogenic variants in ciliopathies associated with retinal degeneration (Potter et al., 2021). Another advancement in the study of retinal architecture came with the application of ExM to the mouse retina, enabling nanoscale resolution of photoreceptor substructures. In 2022, our group leveraged this approach to uncover a previously unrecognized structural feature within the connecting cilium, termed the inner scaffold, which maintains cohesion of microtubule doublets (Mercey et al., 2022). Mutations of FAM161A, an inner scaffold component associated with retinitis pigmentosa, result in microtubule doublet disorganization, outer segment collapse and ultimately photoreceptor degeneration. Additionally, ExM has proven valuable for elucidating pathogenic mechanisms in other inherited retinal disorders, including Leber congenital amaurosis (Faber et al., 2023) and retinitis pigmentosa (Mercey et al., 2024), by linking genetic mutations to specific ultrastructural abnormalities.

### Future directions – imaging-based strategies for ciliopathy diagnosis and therapeutic monitoring

#### Imaging approaches for ciliopathy diagnosis

Despite advances in genomics, a significant proportion of ciliopathies still lack a definitive molecular diagnosis. For instance, although over 50 genes have been associated with PCD, up to 30% of PCD cases remain without an identified genetic cause (Dodd et al., 2024). This diagnostic gap underscores the need for complementary approaches, among which advanced imaging modalities seem promising. In this context Liu and colleagues developed a quantitative imaging workflow to analyze patient nasal airway cells using SIM and STORM (Liu et al., 2020). These advanced imaging methods enabled the quantitative assessment of mislocalization of a panel of PCD proteins, the detection of structural abnormalities and the measurement of ciliary motility defects. Looking ahead, this approach could be extended to anatomically inaccessible tissues (such as the retina) by leveraging induced pluripotent stem cell (iPSC) organoids derived from individuals with ciliopathies. Such models would not only facilitate patient-specific diagnosis but also support the development of personalized therapeutic strategies. Additionally, a recent study combined next-generation sequencing, proteomics and cryo-EM to elucidate how different variants in the same gene, tubulin β4A (*TUBB4A*), lead to distinct clinical symptoms (Dodd et al., 2024). Using near-atomic resolution imaging, the researchers demonstrated how specific mutations disrupt precise tubulin interfaces, causing functional defects that explain the observed individual phenotypes.

#### Imaging strategies to monitor gene therapy efficacy

Gene therapy holds great promise for treating ciliopathies through restoration of functional gene expression in affected cells. However, significant challenges remain in evaluating the efficacy and precision of these therapeutic interventions at the subcellular level. Traditional outcome measures often rely on global functional rescue or tissue-level readouts, which might fail to capture subtle or incomplete restoration of protein localization. SRM offers a powerful tool to visualize the subcellular distribution of re-expressed proteins within native subcellular structures. These imaging modalities thus serve as sensitive indicators of therapeutic success or failure in preclinical studies. For example, we recently used ExM to fine-tune the re-expression of *Fam161a* in a mouse model of retinitis pigmentosa (Arsenijevic et al., 2024). The nanoscale resolution of this technique enabled us to correlate promoter strength with precise protein localization along the photoreceptor cilium. Importantly, this approach revealed that a threshold level of protein re-expression is necessary to achieve functional restoration of photoreceptor cells. This study opens new avenues for optimizing viral vectors, dosing and isoform selection in preclinical gene therapy development.

#### Imaging tools for visualizing subciliary dynamics

Advanced imaging has transformed ciliopathy research by revealing the intricate architecture of the cilia and localizing disease-related proteins at the nanoscale, uncovering structural and molecular defects. However, most of these methods require fixed samples, limiting analysis to static snapshots and missing dynamic ciliary processes. A key method in this field has been total internal reflection fluorescence (TIRF) microscopy, which enables visualization of IFT dynamics and ciliary trafficking (Engel et al., 2009; Stepanek and Pigino, 2016). Yet, its resolution remains limited. To overcome this, emerging super-resolution live-cell imaging technologies, such as MINFLUX nanoscopy, enable the real-time tracking of molecular dynamics with near-molecular precision. Deguchi and colleagues have tracked individual fluorophores with nanometer-scale spatial and millisecond temporal resolution in both 2D and 3D (Deguchi et al., 2023). Using this approach, they resolved the precise stepping behavior of the motor protein kinesin-1 along microtubules within living cells. One year later, Schleske and colleagues extended this technique to track dynein motors in primary neurons (Schleske et al., 2024). Applied to cilia, MINFLUX could elucidate IFT by tracking IFT proteins along axonemal microtubules, distinguishing transport directions and cargo events. It could also reveal ciliary signaling dynamics, including receptor trafficking and molecular clustering, thereby clarifying how spatial organization drives signal transduction in health and disease. With such tools, one can now uncover how disease-associated mutations alter molecular behaviors in real time. Moreover, it is now feasible to assess whether candidate therapies restore normal dynamics at the level of cellular organelles. Thus, dynamic nanoscale imaging of cilia would not only deepen mechanistic understanding of ciliopathies but would also help therapeutic evaluation.

## Conclusions and perspectives

The chronological progression of imaging modalities, from classical TEM to super-resolution fluorescence microscopy and cryo-EM, has consistently pushed the frontier of imaging for cilia and centrioles, enabling not only the visualization but also the mechanistic interpretation of how centriolar and ciliary structures function in health and disease. Electron microscopy, and notably cryo-EM, has revealed structural details at near-atomic resolution, redefining models of axonemal architecture. Complementing EM approaches, super-resolution light microscopy techniques such as STED, STORM, SIM and ExM have bridged the gap between ultrastructure and protein localization, shaping the concept of molecular architecture. As imaging technologies continue to evolve, particularly with the integration of AI-driven image analysis and *in situ* cryo-ET, we are poised to gain even deeper insights into the nanoscale biology of centrioles and cilia, and their roles in an expanding array of human diseases.

Looking ahead, the integration of these technologies into tissue imaging and live imaging represents crucial challenges. Applying advanced modalities in native tissue contexts will clarify how centrioles and cilia contribute to organ development and function, while live imaging will enable dynamic tracking of structural and signaling changes in real time. These approaches hold promise not only for basic discovery but also for translational applications, from assessing ciliary dysfunction in disease to monitoring therapeutic options in the context of ciliopathies.
